# Sugar content and nutrient content claims of growing‐up milks in Indonesia

**DOI:** 10.1111/mcn.13186

**Published:** 2021-04-08

**Authors:** Alissa M. Pries, Anzélle Mulder, Jane Badham, Lara Sweet, Katelyn Yuen, Elizabeth Zehner

**Affiliations:** ^1^ Helen Keller International Headquarters Washington District of Columbia USA; ^2^ JB Consultancy Johannesburg South Africa

**Keywords:** breast‐milk substitutes, growing‐up milk, infant and young child nutrition, sugar, sugar‐sweetened beverages, toddler milk

## Abstract

‘Growing‐up milks’ (GUMs)—breast‐milk substitutes targeted for young children (aged 12–36 months)—are commonly consumed in Indonesia. The World Health Assembly has stated that GUMs are not necessary for proper growth and development, and recently, the American Academy of Pediatrics declared that such products are not recommended for young children due to their common use of sweeteners. To contribute to the evidence base on the composition of GUMs and their appropriateness for young child diets, this cross‐sectional study documented the declared sugar content and presence of nutrient content claims of 99 GUMs newly launched in Indonesia between January 2017 and May 2019. Sugar content was evaluated against the draft 2018 Codex Alimentarius Standard for Follow‐Up Formula guidance on sugar content and the United Kingdom Food Standard Agency's front‐of‐pack (UK FSA FOP) colour coding system for sugar. Almost all (97%) GUMs contained one or more added sugars. None of the products were fully compliant with all three sugar content recommendations in the draft Codex standard. Seventy‐one per cent of GUMs were determined to have high sugar content according to the UK FSA FOP system. Nutrient content claims were found on 97% of GUMs. Median total sugar content was 7.3 g per 100 ml, similar to sugar content levels in sugar‐sweetened beverages. Many GUMs available in Indonesia claim to offer nutritional benefits; however, the current levels of sugar content in GUMs are a serious concern and are inappropriate for inclusion in the diets of young children.

**Key messages**

- Almost all GUMs contained one or more added sugars and had sugar content in excess of global recommendations, making them inappropriate for young children.
- Despite high sugar content, GUMs made extensive use of nutrient content claims on their labels.
- Considering their inappropriately high sugar content, without a national front‐of‐pack labelling system to warn caregivers, many GUMs may appear to be nutritionally suitable for young children.
- To protect young children, Codex must complete the work on revising the Codex Standard on Follow‐Up Formula, and national laws related to GUMs must align with global standards in limiting added sugar and restricting total sugar content.

## 1 INTRODUCTION

The World Health Organization (WHO) recommends exclusive breastfeeding until 6 months of age, at which time appropriate complementary foods should be introduced and breastfeeding continued to 2 years of age or beyond (WHO & UNICEF, 2003). Optimal and continued breastfeeding has been shown to reduce the risk of child morbidity and mortality (Sankar et al., 2015) and is also associated with reduced risk of childhood obesity (Yan et al., 2014). With only half of all Indonesian children continuing to receive the benefits of breastfeeding at 2 years of age (National Population and Family Planning Board et al., 2018), there is a need to protect and improve continued breastfeeding practices in Indonesia. Consumption of breast‐milk substitutes is prevalent among Indonesian infants and young children. As of 2017, nearly three quarters (72.9%) of non‐breastfeeding children 6–23 months of age were consuming a breast‐milk substitute (National Population and Family Planning Board et al., 2018). A recent health facility‐based survey in Bandung City, Indonesia, identified that half of 12‐ to 35‐month‐olds had consumed a breast‐milk substitute in the previous day (Helen Keller International, 2021), and a survey among mothers living in Jakarta found that GUMs were consumed seven or more times per week by nearly one third of young children 12–35 months of age (Willcox et al., 2021).

Breast‐milk substitutes targeted for young children (defined as children 12–36 months of age), often referred to as ‘growing‐up milks’ (GUMs) or ‘toddler milks’, have become an increasingly prominent source of revenue for breast‐milk substitute manufacturers (Rollins et al., 2016). In Indonesia, increasing rates of breast‐milk substitute use among young children are driven primarily by rising use of GUMs, with GUM sales increasing from 12.3 billion IDR to 24.0 billion IDR between 2011 and 2016 and an anticipated 23% value growth for GUMs in Indonesia between 2016 and 2021 (Euromonitor International, 2016). GUMs are often marketed as beneficial to young child development, with labels making nutrient content claims and claims around growth and health that may persuade caregivers that these products are essential for young children (Champeny et al., 2019; Pereira et al., 2016; Pomeranz, Romo, & Harris, 2018). However, GUMs are considered an ultra‐processed food (Monteiro et al., 2017) and consist of primarily powdered milk, corn syrup solids or other added sugars, and vegetable oil (Pomeranz et al., 2018). GUMs tend to have higher energy density and contain more added sugars than infant formulas and follow‐up formulas (Koletzko et al., 2013) and commonly use sweet flavourings that appeal to young children (Harris et al., 2016), potentially making them another avenue for establishing sweet taste preferences early in life and caregiver reliance on sweetened beverages for young child feeding (Park et al., 2014). A recent study found that GUMs available in Australia had higher sugar content than cow's milk (McCann et al., 2020). Research has established a relationship between sweet drink consumption and increased risk for overweight and obesity among children (Malik et al., 2013), including among young children under 36 months of age (Welsh et al., 2005). Consumption of sugar‐sweetened beverages is prevalent among young children in Indonesia (Green et al., 2019), and childhood obesity is rising, with approximately one in 15 school‐aged children overweight (Oddo et al., 2019).

Despite being marketed as healthy and nutritionally beneficial, the appropriateness of GUMs for young children's diets has been questioned for over three decades. As far back as 1986, the World Health Assembly (WHA resolution 39.28) agreed that these milks targeted for older infants and young children were not necessary for young child nutrition and health. Furthermore, a 2010 WHA resolution (WHA 63.23) agreed that nutrition claims should not be made on foods and beverages for infants and young children, including GUMs, unless provided for by Codex Alimentarius (Codex) or national legislation. In the guidance included as part of the 2016 resolution (WHA 69.9), GUMs were once again recognized as functioning as breast‐milk substitutes and therefore covered by the WHO International Code of Marketing of Breast‐milk Substitutes, which states that there should be no advertising or any form of promotion for these products (WHO, 1981, 2017). As recently as 2019, an expert panel, including the American Academy of Pediatrics and the Academy of Nutrition and Dietetics, voiced that GUMs are nutritionally unnecessary and not recommended for children 12 months and older, as well as noting their concerns of caloric sweeteners used in these products (Lott et al., 2019). Evidence regarding the appropriateness of the nutritional composition and sugar content of GUMs would be useful to assist national governments in establishing their own regulations and standards to ensure appropriate nutrition for young children who consume GUMs.

The aim of this study was to assess sugar levels of GUMs available on the Indonesian market to document the appropriateness of such products for inclusion in diets of young Indonesian children. Using label information from cow's milk‐based GUM products that came onto the Indonesian market between January 2017 and May 2019, this study assessed total sugar content, types of sugars, sweeteners and flavourants included in GUM composition and their appropriateness for inclusion in young child diets based on these factors. Nutrient content claims made on the labels of these products were also assessed. The objectives were as follows:

1. to assess the sugar level of GUMs, including presence and types of added sugars;
2. to assess the presence and types of sweeteners and flavourings added to GUMs;
3. to evaluate GUMs for compliance with draft 2018 Codex Standard for Follow‐Up Formula guidance on sugar content;
4. to evaluate sugar content based on the United Kingdom Food Standard Agency's (UK FSA) front‐of‐pack (FOP) colour coding system; and
5. to determine the proportion of GUMs presenting nutrient content claims on their labels, the nutrients being claimed, and to assess the proportion of products with nutrient content claims that would require ‘high sugar’ (red) according to the UK FSA FOP colour coding system.

## 2 METHODS

### 2.1 Study design and data source

This study involved a cross‐sectional assessment of label information declared on new GUM products newly launched in Indonesia over a 28‐month period. For the purposes of this study, GUMs were defined as ‘growing‐up milks’, ‘toddler milks’ and similar products intended for young children aged 12–36 months. This study included only cow's milk‐based drinks (either in liquid form or in powder form to be reconstituted), with or without modification of the protein composition or content and supplementation of fatty acids, micronutrients or other substances with a potential nutritional effect, such as probiotics, prebiotics or symbiotics (European Food Safety Authority, 2013).

A database of GUMs launched in different cities across Indonesia between January 2017 and May 2019 was purchased from Innova Market Insights (IMI), a market research company. This database included only newly launched products and did not include pre‐existing products in Indonesia. This timeframe was obtained to cover two full calendar years (2017 and 2018) and additional months preceding data purchase. Such third‐party sales data are typically objective and reliable and can provide comprehensive data on availability of food products across time (WHO Regional Office for Europe, 2020). These newly launched products were identified by Innova network members who conducted weekly (at a minimum) visits to different retailers of various channels in cities across Indonesia. After new product identification, label text information for each product was extracted by IMI, entered into their database, and label information in Bahasa Indonesia was translated to English. For quality control of label information extraction, product records were checked by local and regional IMI editors.

The GUMs database was received from IMI as a Microsoft Excel spreadsheet. The following data were included: product purchase date, product identifiers (e.g., manufacturer and brand name) and packaging information (e.g., package size and material), and all information present on labels. For this study, the following label information was used in the analysis: ingredients list, nutritional information per 100‐g powder or per 100 ml for ready‐to‐drink GUMs, nutrient content claims, age range for use, serving size information and recommended number of servings. Sugar content in reconstituted values per 100 ml and per serving was calculated (or captured for GUMs in liquid form). All information provided in the label's nutrient declaration regarding sugar content, in whichever form, was captured (e.g., total sugar, added/free sugar, sucrose and lactose).

For this label assessment, products were excluded from analysis based on the following criteria: (1) if label information was not originally in Bahasa Indonesia or English; (2) if products were not cow's milk based; and (3) if products were solely for special medical purposes and therefore did not meet the GUM study definition.

### 2.2 Data management and analysis

The sugar content and types of sugars added to GUMs were assessed based on a review of the ingredient lists and nutrition information declarations provided on their labels. Added sugars are defined by the WHO as all monosaccharides and disaccharides added to products by the manufacturer, cook or consumer, and sugars naturally present in honey, syrups, fruit juices and fruit juice concentrates (WHO, 2015). The lactose naturally present in the dairy‐based component of products is not included in the definition of added sugars, unless the addition of lactose by the manufacturer is noted in the ingredient list (Swan et al., 2018). Product ingredient lists were also reviewed to identify the presence and types of sweeteners and flavourants added to products.

Compliance of GUM products with the Codex Alimentarius 2018 Draft Revised Standard for Follow‐Up Formula (CXS 156‐1987) (referred hereafter as ‘draft 2018 Codex Standard’) was evaluated (Codex Alimentarius, 2018). The Codex Committee on Nutrition and Foods for Special Dietary Uses (CCNFSDU) agreed that the standard, originally issued in 1987, was outdated and began a full review of the standard in 2015 and is ongoing. The version of the revised text used for this study was that agreed at the conclusion of the 2018 CCNFSDU meeting. It is an opportune time to contribute to the evidence base on the appropriate composition of products for young children by assessing how GUMs already on the market perform against this draft standard. This draft 2018 Codex Standard is composed of two sections, one for products intended for older infants 6–12 months of age and one for products intended for young children 12–36 months of age; recommendations in the latter section were referenced for this analysis. The draft 2018 Codex Standard provides global composition recommendations for these types of products for young children (12–36 months). Three recommendations relevant to sugar content were used for this evaluation.

**FIGURE 1:**
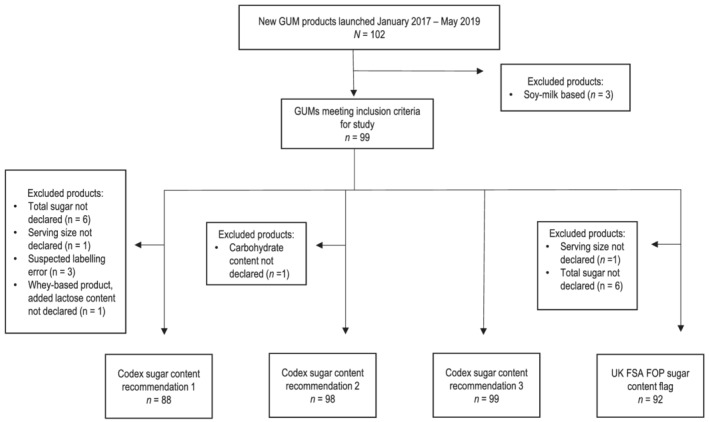
Growing‐up milk (GUM) product exclusion for sugar content evaluation. UK FSA FOP, United Kingdom Food Standard Agency's front‐of‐pack

First, the draft 2018 Codex Standard requires that monosaccharides and disaccharides should not exceed 2.5 g per 100 kcal (0.60 g per 100 kJ); this does not include lactose, naturally occurring or added. It also states that national and/or regional authorities may further limit this level to 1.25 g/100 kcal (0.30 g/100 kJ). Total monosaccharide and disaccharide content of GUMs was assessed based on the nutrition information provided on the labels. If monosaccharide and disaccharide content was not provided on the label, this was calculated by subtracting the declared lactose composition of the product from the total sugar content per 100 g of reconstituted/ready‐to‐drink product. If the lactose composition of the product was not provided, a standard value of lactose content in cow's milk (5.1 g per 100 g) was used. The lactose composition of cow's milk per 100 g was calculated as an average of the lactose composition of non‐fortified whole cow milk, 2% fat cow milk, 1% fat cow milk and non‐fat cow milk (US Department of Agriculture, 2015). A standard lactose value was used for 25 of the 99 products. For three products, the subtraction of this standard value of lactose resulted in a negative remaining sugar content. For these products, total sugar was between 0 and 2 g per 100 g of product and carbohydrate content was between 5 and 10 g per 100 g of product; given that ingredients in these products were primarily dairy products and sugar, nearly all carbohydrate content should be sugars (lactose and other monosaccharides and disaccharides). It was suspected that these three products had labelling errors, and they were therefore excluded from analysis (Figure 1). For one product, the GUM was cow's milk based but noted whey protein in the ingredients and inherent lactose would therefore be minimal, but it also listed added lactose as an ingredient, but total lactose content was not declared. Because the standard value of inherent lactose would not apply to this product, but the amount of added lactose was not known, this product was also excluded from this analysis (Figure 1). Products were classified as prudent, compliant or non‐compliant according to their monosaccharide and disaccharide (excluding lactose) content: prudent, ≤1.25 g/100 kcal (0.3 g/100 kJ); compliant, >1.25 to ≤2.5 g/100 kcal (>0.3 to ≤0.6 g/100 kJ); and non‐compliant, >2.5 g/100 kcal (>0.6 g/100 kJ).

Second, the draft 2018 Codex Standard sets an upper limit of 12.5 g of carbohydrate/100 kcal (3.0 g/100 kJ) as an additional precautionary measure to limit sugar in these products. GUMs that exceeded this threshold were classified as ‘non‐compliant’. For GUMs with a protein level below 3.0 g/100 kcal, the draft 2018 Codex Standard states that a maximum level of available carbohydrates up to 14 g/100 kcal (3.3 g/100 kJ) may be permitted by competent national and/or regional authorities. Relevant products were also assessed for compliance against this more lenient threshold, and changes in compliance status noted. For the first and second recommendations, the draft 2018 Codex Standard is intended for products ‘prepared ready for consumption in accordance with the instructions of the manufacturer’. Therefore, total sugar, lactose and carbohydrate contents were calculated for reconstituted products following reconstitution instructions on the label. One product did not provide carbohydrate content information and was excluded from this analysis (Figure 1).

Third, the draft 2018 Codex Standard states that sucrose and/or fructose should not be added. Ingredient lists were reviewed for the addition of sucrose or fructose to GUMs, and products with the presence of either in their ingredient lists were classified as ‘non‐compliant’.

Total sugar content of GUMs was assessed using the UK FSA FOP colour coding system. The UK FSA FOP system was used in this study to characterize the degree of total sugar present in the GUMs to further understand the appropriateness of including these products in the diets of young children. Rather than assessing sugar content based on a threshold, as set out in the draft Codex standards, the UK FSA FOP system assesses the degree of sugar content, which is a useful additional evaluation for GUMs. In addition, because the Codex standard is currently under review, it is useful to compare against another set of criteria that have been fully implemented, such as the UK FSA FOP system. Finally, we sought to assess whether GUMs would warrant an FOP label to inform consumers of the high presence of negative nutrients, such as sugar content. Given that such a system does not exist in Indonesia, the UK FSA FOP system was used as an example FOP system for this evaluation. In this system, sugar content of a product is evaluated, and a colour assigned accordingly. Beverage products are assigned green (low sugar level) if total sugar is ≤2.5 g/100 ml, orange (medium level) if total sugar is >2.5 to ≤11.25 g/100 ml or red (high level) if total sugar is >11.25 g/100 ml and the serving size is less than 150 ml or if total sugar is >13.5 g/portion if the serving size is greater than 150 ml (United Kingdom Department of Health & Food Standards Agency, 2016). In cases where serving sizes were listed per weight and not per volume, nutrient values per 100 g of GUM were converted to values per 100 ml using the density of 1.03 g/cc (100 ml of milk equals 103 g), which is the density of both cow's milk and reconstituted infant formula (AVCalc LLC, 2019). Six products did not provide total sugar content information, and one did not provide serving size information, and so these seven products were excluded from this analysis (Figure 1).

The presence of nutrient content claims on the labels was assessed by reviewing label information for statements/images that described the level of a micronutrient or macronutrient contained in the product such as ‘source of (nutrient)’, ‘high in (nutrient)’, ‘very high in (nutrient)’, ‘low in (nutrient)’, ‘very low in (nutrient)’ or ‘free from (nutrient)’ (Codex Alimentarius, 2013). GUMs were categorized as either having or not having a nutrient content claim, with the types of nutrients being claimed noted.

Data were analysed in Stata 14 (Stata Corp, College Park, TX, USA). Compliance with each draft Codex standard recommendation and performance in the UK FSA FOP colour coding system was calculated for each product, and prevalence rates were summarized using proportions. Medians with interquartile ranges (IQRs) were used to present descriptive statistics for non‐normally distributed data. Differences in proportions of products were tested using a Pearson chi‐square test, and differences in medians were tested using a Kruskal–Wallis test, with significance defined as *p* < 0.05. Products that did not provide sufficient label information required to assess specific objectives were excluded from relevant analyses and have been noted.

## 3 RESULTS

The database provided by IMI initially included 102 GUMs. Three GUMs were excluded because they were soymilk based. No products were excluded because of label language or because they were for special medical purposes. The final analysis included 99 GUMs. Figure 1 provides details on product exclusion from the study and from each sugar content evaluation. Just over two thirds of the GUMs were from international manufacturers (68.7%, *n* = 68), whereas one third were from national Indonesian manufacturers (Table S1). Products were most commonly manufactured by Nutricia and Nestlé, which each made up approximately one quarter (24.2%, *n* = 24) and one fifth (21.2%, *n* = 21), respectively, of all GUMs. The majority of GUMs were powdered products requiring reconstitution, whereas nine products (9.1%) were ready‐to‐drink.

### 3.1 Added sugar, sweeteners and flavourants

Nearly all GUMs (97.0%, *n* = 96) contained one or more added sugars. These included sucrose, fructose or other sugars according to the information provided in the ingredient list (Table 1). Sucrose, lactose, honey derivatives and solid glucose syrup were the most common sugars added to GUMs. GUMs with added sugars contained a range of 1–5 different added sugars, with a median of 2.

**TABLE 1:** Types of added sugars listed in ingredients of GUMs (*n* = 99)

Added sugar	% (*n*)
**GUMs with added sugar**	**97.0% (96)**
Sucrose	76.8% (76)
Lactose	70.7% (70)
Any honey derivatives	31.3% (31)
Honey powder	20.2% (20)
Honey	11.1% (11)
Natural honeycomb	3.0% (3)
Solid glucose syrup	22.2% (22)
Fructose	4.0% (4)
Solid corn syrup	3.0% (3)
Sugar	1.0% (1)

Abbreviation: GUMs, growing‐up milks.

Beyond sugars, other non‐nutritive sweeteners were also commonly added to the GUM products, including oligosaccharides (35.4%, *n* = 35), inositol (11.1%, *n* = 11), polyfructose (5.1%, *n* = 5) and isomaltulose (2.0%, *n* = 2). In total, 44 GUM products contained added non‐sugar sweeteners, with all but one of these products also containing added sugar. The majority of GUMs (83.8%, *n* = 83) were flavoured and included vanilla (37.1%, *n* = 36), honey (36.1%, *n* = 35), chocolate (7.2%, *n* = 7), strawberry (4.1%, *n* = 4) and fruity (1.0%, *n* = 1) flavours. Median total sugar content per 100 ml of GUM was significantly higher among flavoured GUMs as compared with non‐flavoured GUMs (7.5 g vs. 5.9 g per 100 ml, respectively, *p* = 0.016).

### 3.2 Evaluation of GUM products for compliance with draft 2018 Codex Standard for Follow‐Up Formula guidance on sugar content

The first draft 2018 Codex Standard recommendation assessed monosaccharide and disaccharide (excluding lactose) content per 100 kcal of GUMs (Table 2). Among the 88 products that could be analysed for compliance with the first draft 2018 Codex Standard monosaccharide and disaccharide recommendations, median total monosaccharide and disaccharide content per 100 kcal was 2.5 g (IQR: 1.5–3.4 g), which is the cut‐off quantity for compliance with this draft 2018 Codex Standard recommendation. Over one third of products were non‐compliant with the draft 2018 Codex Standard (38.6%, *n* = 34/88), whereas 38.6% (*n* = 34/88) were compliant and 22.7% (*n* = 20/88) were prudent. Nearly three quarters of international products that could be assessed against this draft 2018 Codex Standard recommendation (71.0%, *n* = 44/62) were compliant, as compared with 38.5% (*n* = 10/26) of national products (*p* = 0.004).

**TABLE 2:** Evaluation of growing‐up milk products against draft 2018 Codex Standard and UK FSA FOP flags for sugar content

Evaluation criterion	Products that could be assessed based on label information	% (*n*)
**Draft 2018 Codex Standard recommendations for sugar content**		
Recommendation 1: total monosaccharide and disaccharide content ≤ 2.5 g/100 kcal	88	61.4 (54)
Recommendation 2: <12.5 g of carbohydrate/100 kcal	98	6.1 (6)
Recommendation 3: no added fructose or sucrose	99	23.2 (23)
**UK FSA FOP sugar content flags** ^a^		
Low sugar (green flag)	92	4.4 (4)
Medium sugar (orange flag)	92	25.0 (23)
High sugar (red flag)	92	70.6 (65)

Abbreviation: UK FSA FOP, United Kingdom Food Standard Agency's front‐of‐pack.

^a^ Green flag: total sugar content ≤ 2.5 g/100 ml; orange flag: total sugar content > 2.5 g to ≤11.25 g/100 ml; and red flag: total sugar content > 11.25 g/100 ml and serving size < 150 ml or total sugar content > 13.5 g/portion and serving size > 150 ml.

The second draft 2018 Codex Standard recommendation assessed carbohydrate content per 100 kcal of GUM products (Table 2). Among the 98 GUMs that did provide carbohydrate content information, median carbohydrate content per 100 kcal was 14.0 g (IQR: 13.1–14.7 g), which exceeds the cut‐off quantity for compliance with this draft 2018 Codex Standard recommendation. The vast majority of these products (93.9%, *n* = 92) were found to be non‐compliant with the draft 2018 Codex Standard recommendation for maximum carbohydrate content, whereas six products (6.1%) were compliant. Compliance increased to seven products (7.1%) when the higher cut‐off quantity that may be permitted by competent national and/or regional authorities was applied to products with less than 3 g of protein per 100 kcal (*n* = 8). A greater proportion of national GUM products (16.7%, *n* = 5/30) were compliant with this second draft 2018 Codex Standard recommendation, as compared with international products (1.5%, *n* = 1/68) (*p* = 0.004).

The third draft 2018 Codex Standard recommendation for sugar content assessed the presence of added fructose or sucrose in GUMs (Table 2). Three quarters of all GUMs (76.8%, *n* = 76) contained either added fructose or sucrose and were therefore non‐compliant. The majority of these products were non‐compliant due to the addition of sucrose, with 72 products (72.7%) containing added sucrose (no fructose) and four products (4.0%) containing both added sucrose and fructose. The majority of both national (87.1, *n* = 27/31) and international products (72.1%, *n* = 49/68) did not comply with this third draft 2018 Codex Standard recommendation (*p* = 0.100). Of all 99 GUMs, no products were compliant across all three draft 2018 Codex Standard recommendations for sugar content.

### 3.3 Evaluation of GUM products against UK FSA FOP sugar content flags

Total sugar content per 100 ml of GUM products was assessed to evaluate the products against the UK FSA FOP colour coding system for sugar (Table 2). Across the 92 products included in this analysis, median total sugar content was 7.3 g per 100 ml of reconstituted/ready‐to‐drink product (IQR: 6.2–7.8 g per 100 ml) and median total sugar content per serving was 15.0 g (IQR: 13.0–19.0 g per serving). Of the 92 GUM products that could be evaluated, the majority (70.6%, *n* = 65) had high total sugar content (>11.25 g/100 ml or >13.5 g/portion) that would warrant a red FOP flag. Only four products (4.4%) had low sugar contents (≤2.5 g/100 ml) that would allow for a green FOP flag. A significantly larger proportion of international products were flagged for high sugar (81.0%, *n* = 51/63), as compared with national products (48.3%, *n* = 14/29) (*p* = 0.001).

### 3.4 Nutrient content claims on GUM products

Almost all (97.0%, *n* = 96) of the 99 GUMs assessed presented a nutrient content claim on their label. GUM products were found to make ‘source of’ claims most frequently for inulin (18.2%, *n* = 18), vitamin A (17.2%, *n* = 17), vitamin B2 (16.2%, *n* = 16), vitamin D (16.2%, *n* = 16) and vitamin E (16.2%, *n* = 16). ‘High in/rich in’ claims were most commonly used for zinc (18.2%, *n* = 18), vitamin A (14.1%, *n* = 14), vitamin C (14.1%, *n* = 14), calcium (14.1%, *n* = 14) and vitamin E (12.1%, *n* = 12). Only one product (1.0%) had a ‘low in’ claim related to sucrose content.

Of the 89 products (89.9%) that displayed nutrient content claims and also declared total sugar content, over two thirds (69.7%, *n* = 62) were red flagged for their high sugar content. The one product that claimed ‘low in sucrose’ was categorized as high in total sugar content and had 19 g of sugar per 228 ml of serving.

## 4 DISCUSSION

This study assessed label information of 99 GUMs launched on the Indonesian market between January 2017 and May 2019. To our knowledge, this is the first study to comprehensively assess GUM sugar content in one of the fastest growing markets, Southeast Asia, for these products (Baker et al., 2020). Nearly all GUMs (97.0%, *n* = 96) assessed contained added sugar, and most had sugar content in excess of draft 2018 Codex and UK FSA FOP recommendations. Indonesian legislation allows for nutrient content claims to be made on GUMs, and nearly all the products in this study made such claims. As nutrient content claims on labels are intended to alert consumers to their purported nutritional benefits, the high sugar content found in these products intended for young children 1–3 years of age and the rapidly expanding market for these products makes the inclusion of nutrient content claims a serious public health concern. These results provide further evidence of the inappropriateness of these products and their nutrient content claims for such young consumers.

Added sugar was found in the ingredient lists of 96 of the 99 (97.0%) GUM products assessed in this study, and most products were further sweetened with non‐nutritive sweeteners or sweet flavourants, with this sweetness undoubtedly aimed to appeal to young children's taste preferences (Harris et al., 2016). Median total sugar content across the GUMs available in Indonesia was 7.3 g per 100 ml. In comparison, total sugar content of full fat cow's milk is approximately 5 g per 100 ml, whereas sugar‐sweetened beverages commonly consumed by young children in Indonesia, including strawberry‐ and chocolate‐flavoured milk and sweet tea products (Green et al., 2019), contain 6.4, 6.8 and 7.2 g of sugar per 100 ml, respectively, according to their labels and product website (Coca‐Cola Company, 2018). A recent assessment of Australian GUMs found that sugar content levels were similar to Fanta available in Australia, which contains approximately 8 g of sugar per 100 ml (McCann et al., 2020). Such findings indicate that GUMs are providing young children with sugar content similar to sugar‐sweetened beverages rather than that of cow's milk, which is recommended for children over 1 year of age. Prior research indicates that the total sugar content of GUMs available in Indonesia is similar to trends among breast‐milk substitutes in other parts of the world. One study assessing 32 toddler milks on the Australian market found that 90% of products contained added sugar, with a mean total sugar content of 7.1 g per 100 ml (McCann et al., 2020). Another study reported that 80% of infant formulas available in the United States were found to contain added sugar (Walker & Goran, 2015). An assessment of breast‐milk substitutes on the market across 11 countries (Brazil, Cambodia, Canada, Columbia, France, New Zealand, Sudan, Spain, Switzerland, the United Kingdom and the United States) reported an average total sugar content of 7.0 g per 100 ml among products aimed at young children 12 months and older (Bridge et al., 2020). Young children's exposure to sugars can increase their risk of early childhood dental caries, particularly when consumed in a bottle such as GUMs typically are (Tungare & Paranipe, 2019). Exposure to sugars and sweeteners early on in life, particularly through sugar‐sweetened beverages, has also been shown to establish taste preferences for sweet foods/beverages that continues throughout childhood (Park et al., 2014) and can present increased risk for childhood obesity (Jimenez‐Cruz et al., 2010).

The majority of GUMs launched in Indonesia over a 28‐month period not only contained added sugars but also were found to have inappropriately high levels of sugar when compared with the global sugar content recommendations in the draft 2018 Codex Standard and also the UK FSA FOP colour coding system. None of the products met all three recommendations in the draft 2018 Codex Standard, with three quarters of products containing either sucrose or fructose and one third of products having excessive monosaccharide or disaccharide content. Although Indonesia does not yet have national policy to evaluate sugar content for consumer awareness using an FOP labelling system, our analysis found that three out of four GUM products launched in Indonesia contained such high sugar levels that they would warrant a red warning label in the United Kingdom. Significant product reformulation of GUMs is required for them to conform with sugar recommendations set in the draft 2018 Codex Standard and the UK standard for FOP labelling.

The lack of compliance with the draft 2018 Codex Standard recommendations for sugar content highlights not only the importance of these standards for ensuring appropriate products for young children globally but also the importance of ensuring that the full suite of recommendations is taken up in national regulations. When each of the three recommendations is considered individually, our analysis indicates that the evaluation of sugar content in the draft 2018 Codex Standard was more lenient. For example, two thirds of GUMs were compliant with the first Codex recommendation around monosaccharide and disaccharide content; however, almost three quarters (72.2%, *n* = 39/54) of GUMs compliant with this recommendation were found to have high total sugar content based on the UK FSA FOP evaluation. The conflicting results in these two approaches to evaluating sugar content are likely related to the exclusion of added lactose from consideration in this first draft 2018 Codex Standard recommendation. Added lactose was the second most commonly added sugar in GUMs, added to 70.7% of products, contributing to these products' high sugar content and highly sweetened flavour. However, the second draft 2018 Codex Standard recommendation considers overall carbohydrate content of GUMs, as a means to limit overall sugar content of these products. The vast majority of carbohydrates in cow's milk‐based GUMs would be sugars, and the recommended limit of 12.5 g of carbohydrate per 100 kcal covers all sugars, including inherent and added lactose, thereby providing a necessary check on the amount of sugars added to GUMs. It is vital that the CCNFSDU completes their work in strengthening and finalizing these draft standards in order for national authorities to then ensure that their own standards are fully aligned with Codex and protect young children from concerningly high levels of sugar consumption associated with the inclusion of these products in their diets.

The GUM products assessed in this study made extensive use of nutrient content claims, with 97.0% (*n* = 96) of products displaying such a claim on their label. The WHO International Code of Marketing of Breast‐milk Substitutes prohibits nutrition claims on breast‐milk substitutes, including GUMs (WHO, 1981). Based on WHA 63.23 and Codex, they are also not permitted on foods (including GUMs) for the age group 6–36 months, unless specifically provided for in national legislation. According to regulation no. 13/2016—Monitoring Claims on Processed Food Labels and Advertisements—of the Head of the Drug and Food Control Agency (*Badan Pengawas Obat Dan Makanan* [BPOM]), nutrient content claims, provided they meet specific criteria, are currently permitted in Indonesia for foods for young children aged 1–3 years (Peraturan Kepala Badan Pengawas Obat Dan Makanan [PKBPOM], 2016). The presence of nutrient content claims is common on GUM labels; a recent study in Australia found that 72% of toddler milks displayed a nutrient content claim, with an average of four claims per label (McCann et al., 2020), and a review of 17 toddler milk drinks available in the United States found that all displayed at least one nutrient/ingredient claim (Pomeranz et al., 2018). While no prior study has assessed the presence of nutrient content claims on GUM product labels available in an East/Southeast Asian context, a review of parenting magazines in Taiwan also found that 85% of breast‐milk substitute advertisements used nutrient content claims, with most products targeted for young children over the age of 1 year (Chen et al., 2015). Such claims are used by manufacturers to increase sales by indicating to consumers that the product offers a nutritional benefit for their young child, regardless of the total nutrient composition of the product. In one US study, caregivers of young children were shown GUM packages and asked to describe what the messages on the label meant to them; half of caregivers reported that the product provides nutrition that their young child would not receive elsewhere in their diet, and one third said that the product is necessary for their young child to obtain the correct nutrition (Romo‐Palafox et al., 2019). A survey among mothers of 12‐ to 36‐month‐olds in the United States found a strong correlation between mothers' agreement with nutritional marketing claims for toddler milks and their provision of these drinks to their own young child (Romo‐Palafox et al., 2020). A recent survey among mothers of young children in Bandung City, Indonesia, found that mothers who fed their young child a GUM reported that perceived growth and health benefits were important factors for their decision to feed this product (Helen Keller International, 2021), and a qualitative study noted that caregivers of Indonesian young children reported GUMs to be vital to the health of their child (Martha, Amelia, & Myranti, 2017). Although GUM products are often fortified with micronutrients commonly deficient in young child diets in low‐income countries like Indonesia (Fahmida et al., 2014), the overall nutrient profile of the products cannot be considered appropriate for young children, particularly when considering the growing concerns of childhood obesity in these same contexts (National Population and Family Planning Board et al., 2018). Consumption of sweet foods early in life can establish taste preferences that continue throughout childhood and contribute to consumption of energy‐dense sweet foods (Luque et al., 2018), and consumption of sweet drinks among young children 2–3 years of age has been correlated with increased risk of overweight (Welsh et al., 2005). This study shows that nutrient content claims are being made on products that overall do not have a suitable sugar profile for this age group and may be misleading some caregivers into believing that these products are nutritionally required for their young child's health and development.

Nutrition claims, which include nutrient content claims, can create a halo effect and mislead consumers from understanding less healthy aspects of a product (Abrams et al., 2015; Andrews et al., 2000; Dixon et al., 2011). Given that nearly two out of three GUM products that made a nutrient content claim in this Indonesia study were classified as having high sugar content when assessed using the UK FSA FOP colour coding system, caregivers may perceive these products as being highly nutritious due to the nutrient content claims, despite their high sugar content. The WHO's International Code of Marketing of Breast‐milk Substitutes and subsequent relevant WHA resolutions urge member states to ensure that nutrition claims are not permitted for breast‐milk substitutes, with GUMs being classified as breast‐milk substitutes. Thus, for Code compliance, no claims should be made on these products. However, Codex, in its general guidance on claims, states that nutrition claims on foods for infants and young children should not be permitted, unless specifically provided for in national legislation (Codex Alimentarius, 2013; WHO, 1981). Indonesian regulations permit nutrient content claims, which meet specific criteria, on processed foods for young children aged 1–3 years, including GUMs in this category (PKBPOM, 2016). This goes against the WHO International Code of Marketing of Breast‐milk Substitutes and subsequent WHA resolutions, which classify GUMs as breast‐milk substitutes. In addition, without a national FOP labelling system to alert caregivers of high negative nutrient contents such as sugar, many GUMs available in Indonesia are sold as being suitable for young children, based on their micronutrient profile, despite their inappropriately high sugar content. This is problematic, and to prevent products with an overall nutrient composition that is not considered healthy for young children from misleading caregivers, appropriate nutrient profiling should be mandatory for any product to display nutrient content claims.

Addressing the concerning levels of sugar content in GUMs is particularly important given the burgeoning GUM market and increasing reach of these products into the diets of young children globally (Willcox et al., 2021). Between 2005 and 2019, breast‐milk substitute sales volume increased by 122%, but this was primarily driven by GUMs, which grew by over 220% and contributed nearly half of total sales by volume for breast‐milk substitutes in 2019 (Baker et al., 2020). As market growth plateaus in many high‐income settings, the GUM market growth is being primarily driven by high‐populated middle‐income countries in East and Southeast Asia, including Indonesia (Baker et al., 2020). In 2016, breast‐milk substitute sales in Indonesia reached IDR 34 billion (USD 2.5 billion), doubling in value from the previous 5 years (Euromonitor International, 2016). A study assessing breast‐milk substitute promotions at points of sale in Bandung City, Indonesia, found that GUMs were the most prevalent breast‐milk substitute product type available on the market and were also the most highly promoted in stores (Hadihardjono et al., 2019). With such significant growth and revenue, and with half of Indonesian young children consuming GUM products (Helen Keller International, 2021), it is vitally important for national regulations to align with global standards in order to limit added sugar in these drinks and restrict total sugar contents and to fully comply with the Code that also prohibits their promotion. While many high‐income countries have already moved through the nutrition transition, Indonesia is just entering into a period of growing child overnutrition (National Population and Family Planning Board et al., 2018). Given the contribution of sugar‐sweetened beverages to child overweight and obesity (Malik et al., 2013) and the establishment of taste preferences through early exposure to sweet foods and beverages (Luque et al., 2018), tackling inappropriate sugar levels in products for young children is one preventive measure that must be made to curb this trend.

This study has several limitations. First, the GUM products assessed in this study included those identified by a market research firm as newly launched products and did not include products that already existed on the market. Therefore, this sample of products is not exhaustive of the entire GUM market in Indonesia. However, to our knowledge, the sample of 99 products is larger than any national assessment of GUMs' sugar content conducted previously. A recent study by Bridge et al. (2020) included breast‐milk substitutes available on the market in Cambodia, but only 7 of the 22 Cambodian products were GUMs, and another recent study by McCann et al. (2020) assessed 32 GUMs available on the market in Australia. Additionally, although this study did assess the presence and type of specific nutrient content claims provided on GUM product labels, we did not explore the validity of these claims through laboratory analysis. Finally, in the assessment of the first Codex Standard (monosaccharide and disaccharide content, not including lactose), a standard lactose value per 100 ml based on cow's milk was subtracted from sugar content for products that did not provide lactose content on their label. The true lactose content of these products may be different, particularly for products that contained added lactose.

GUMs available on the market in Indonesia contain concerningly high levels of sugar, often in excess of global recommendations. Added sugar and sweeteners are commonly included in these products, making them inappropriate for consumption by young children. It is recommended that national laws related to GUMs align with global standards in limiting added sugar and restricting total sugar content in order to ensure appropriate young child diets and safeguard their nutrition.

## CONFLICTS OF INTEREST

All authors have no conflicts of interest to declare.

## CONTRIBUTIONS

AM, JB, LS and AMP performed the research. EZ, JB, AM, LS and AMP designed the study. AM, JB, LS and AMP analysed the data. AMP, AM, JB and LS wrote the paper. All authors contributed to review and finalization of the paper.

## Supporting information

**Table S1.** Description of GUM products launched from January 2017 – May 2019 in Indonesia (*n* = 99)

## Data Availability

The data that support the findings of this study are available from Innova Market Insights. Restrictions apply to the availability of these data, which were used under license for this study. Data are available from Innova Market Insights.
