# Supplementary material for: Sugar content and nutrient content claims of growing‐up milks in Indonesia
Source: Matern Child Nutr. 2021 Apr 8;17(4):e13186. doi: 10.1111/mcn.13186 (PMC8476439; doi:10.1111/mcn.13186)
Supplement: Supplementary file 1 — Table S1. Description of GUM products launched from January 2017 – May 2019 in Indonesia (n = 99) [file MCN-17-e13186-s001.docx]

| Supplemental Table 1. Description of GUM products launched from January 2017 – May 2019 in Indonesia (n=99) | | | |
| --- | --- | --- | --- |
| **Manufacturer** | **International or national company** | **Brand** | **% (n)** |
| Nutricia | International | Bebelac | 18.2% (18) |
|  |  | Nutricia | 1.0% (1) |
|  |  | Nutrilon | 5.1% (5) |
| Nestlé | International | Batita | 4.0% (4) |
|  |  | Dancow | 10.1% (10) |
|  |  | Lactogrow | 6.1% (6) |
|  |  | Nan | 1.0% (1) |
| Sarihusada | National | SGM | 14.1% (14) |
| Tempo Scan Pasific | National | Vidoran | 7.1% (7) |
| Frisian Flag | International | Frisian Flag | 5.1% (5) |
| Dumex | International | Dugro | 2.0% (2) |
|  |  | Mamex | 1.0% (1) |
|  |  | Mamil | 1.0% (1) |
| Mead Johnson Nutrition | International | Enfagrow | 3.0% (3) |
|  |  | Sustagen | 1.0% (1) |
| Kalbe Morinaga | National | Chil Go | 1.0% (1) |
|  |  | Chil Kid | 1.0% (1) |
|  |  | Kalbe Morinaga | 1.0% (1) |
| Morinaga | International | Morinaga | 3.0% (3) |
| Ultrajaya Milk | National | Ultrajaya | 1.0% (1) |
|  |  | Ultra Mimi | 2.0% (2) |
| Wyeth | International | S26 | 3.0% (3) |
| Kalbe Farma | International | Kasih | 1.0% (1) |
|  |  | Zee | 1.0% (1) |
| Mirota | National | Lactona | 2.0% (2) |
| Fonterra | International | Anmum | 1.0% (1) |
| Friesland Campina | International | Friso | 1.0% (1) |
| Mirota Ksm | National | Lactona | 1.0% (1) |
| Pt Kalbe Farma | National | Kalbe | 1.0% (1) |
